# The Impact of DJOS Surgery, a High Fat Diet and a Control Diet on the Enzymes of Glucose Metabolism in the Liver and Muscles of Sprague-Dawley Rats

**DOI:** 10.3389/fphys.2019.00571

**Published:** 2019-05-17

**Authors:** Dominika Stygar, Dorian Andrare, Barbara Bażanów, Elżbieta Chełmecka, Tomasz Sawczyn, Bronisława Skrzep-Poloczek, Ewa Olszańska, Konrad Wojciech Karcz, Jerzy Jochem

**Affiliations:** ^1^Department of Physiology in Zabrze, School of Medicine with the Division of Dentistry in Zabrze, Medical University of Silesia, Katowice, Poland; ^2^Clinic of General, Visceral, Transplantation and Vascular Surgery, Hospital of the Ludwig Maximilian University, Munich, Germany; ^3^Department of Pathology, Faculty of Veterinary Medicine, Wrocław University of Environmental and Life Sciences, Wrocław, Poland; ^4^Department of Instrumental Analysis, School of Pharmacy with the Division of Laboratory Medicine in Sosnowiec, Medical University of Silesia, Katowice, Poland

**Keywords:** high fat diet (HF), bariatric surgery, obesity, T2DM, DJOS, glucose metabolism enzymes, dietary patterns

## Abstract

The prevalence of diabetes type 2 (T2DM) and obesity is growing exponentially and becoming a global public health problem. The enzymes of glucose metabolism play a role in the pathogenesis of insulin resistance and T2DM. A pathophysiological link between different dietary patterns, HFD, obesity, T2DM and the enzymes of glucose metabolism can be used as a potential target in therapeutic strategies for the treatment of obesity, and T2DM. The aim of this study was to measure the impact of DJOS bariatric surgery and different types of dietary patterns on glycogen synthase kinase 3 α (GSK-3α), glycogen phosphorylase (PYGM, PYGL), and phosphofructokinase (PFK-1) concentrations in liver and soleus muscle tissues of rats. After 8 weeks on a high-fat diet (HF) or control diet (CD), rats underwent duodenal-jejunal omega switch (DJOS) or SHAM (control) surgery. After surgery, for the next 8 weeks, half of DJOS/SHAM animals were kept on the same diet as before, and half had a changed diet. The concentrations of GSK-3α, PYGM, PYGL and PFK-1 were measured in the soleus muscles and livers of the Sprague-Dawley rats. The type of diet applied before/after surgery had stronger impact on levels of selected metabolic enzymes than DJOS or SHAM surgery. The impact of DJOS surgery was visible for GSK-3α and PYGL concentration in the liver but not in the soleus muscle tissue. The type of bariatric surgery had an impact on liver GSK-3α concentration in all studied groups except the CD/CD group, where the impact of diet was stronger. DJOS bariatric surgery influenced the level of PYGL in the livers of rats maintained on the CD/CD diet but not from other groups. The dietary patterns applied before and after bariatric surgery, had a stronger impact on enzymes’ concentrations than DJOS surgery, and the strong, deleterious effect of an HF was observed. A change of the diet *per se* showed a negative impact on the enzymes’ tissue concentration.

## Introduction

After excessive weight and obesity became widespread, the prevalence of diabetes type 2 (T2DM) grew exponentially and became a global public health problem. It is assessed, that T2DM is the most common form of DM, which accounts for 90–95% of all diabetic patients and is expected to reach 439 million people by the year 2030 (Tripathi and Srivastava, 2006; Chen et al., 2011; Wu et al., 2014). T2DM mostly results from the interaction of the risk factors, which are genetic environmental conditions, type of diet or physical activity. The changes in the physiology of the first-phase of insulin release, impaired pulsatility of basal insulin secretion, and increased glucagon secretion accelerate the development of T2DM. In the pathogenesis of T2D, the insulin resistance leads to impaired response of muscle tissue, liver and adipose tissue to the physiological concentrations of insulin (Kaidanovich-Beilin and Eldar-Finkelman, 2006; MacAulay and Woodgett, 2008). Since a major contributor to T2DM is impaired glucose transport from the blood stream into peripheral tissues, numerous studies implicate the enzymes of glucose metabolism in the pathogenesis of insulin resistance and T2DM. GSK-3 acts as the protein which regulates the activity of glycogen synthase (GS), an enzyme that mediates the conversion of glucose to glycogen (Woodgett and Cohen, 1984; Peat et al., 2004). GSK-3 is known to be a negative regulator of the insulin-signalling pathway, which leads to a decrease of hepatic glycogenesis via the phosphorylation and inactivation of GS. GSK-3 activity is inhibited by high glucose and insulin availability via the action of insulin through PI3 kinase (phosphatidylinositol-4,5-bisphosphate 3-kinase)/PKB (protein kinase B) pathways. Those processes lead to the activation of GS and stimulation of glycogen formation (Frame and Cohen, 2001; Grimes and Jope, 2001; Pandey and DeGrado, 2016). It is known, that over-expression and over-activity of GSK-3 are associated with insulin resistance in type 2 diabetes. During glycogenolysis, breakdown of glycogen to glucose-1-phosphate, which enters glycolysis to fulfil the energetic requirements of the cell, is catalysed by glycogen phosphorylase (GP), a key enzyme for this process (Henke and Sparks, 2006). GP is a tissue specific enzyme, and its isoforms are located within different metabolically active tissues for different physiological functions and are named for the tissues in which they are predominantly expressed: liver, muscle and brain (Newgard et al., 1989). The muscle isoform of GP (PYGM) provides energy for muscle contraction, and the liver isoform (PYGL) regulates glucose release from hepatic glycogen stores (Rath et al., 2000). The 6-phosphofructo-1-kinase (PFK-1) plays a central role in the regulation of glycolysis, catalysing the phosphorylation of fructose 6-phosphate to fructose 1,6-bisphosphate. This is the first point of commitment of glucose to the glycolytic pathway and is essentially irreversible (Weber, 1977; Mor et al., 2011). Duodenal-jejunal omega switch (DJOS) is a bariatric technic variant, which allows for direct hindgut stimulation through bypassing the foregut (Rubino and Gagner, 2002; Nauck, 2009). DJOS procedure allows the pylorus of the patients undergoing surgery to be saved. After this procedure patients do not suffer from conditions such as dumping, diarrhoea or dyspepsia, which are characteristic symptoms of postgastrectomy (Traverso and Longmire, 1978; Grueneberger et al., 2014). DJOS is a relatively recent bariatric technique and as a result an animal model, for studying the physiological processes connected with the short and long-term effect of this procedure is still necessary (Karcz et al., 2013). As a potential target in therapeutic strategies for the treatment of metabolic dysregulation such as obesity, insulin resistance and T2DM, a pathophysiological connection between obesity, different dietary patterns, and enzymes of glucose metabolism may be used. Therefore, the target of the study was to assess the impact of different types of dietary patterns in conjunction with DJOS surgery on GSK-3α, PYGM, PYGL, and PFK-1 in the liver and soleus muscles of rats. It was assumed that, DJOS surgery might have led to decrease in GSK-3α the liver concentration, and in that manner prevent the development of T2DM. A HF and control diet were used as a model similar to human conduct for the exploration of the underlying mechanisms mediating the potential metabolic benefits of DJOS, measured by the concentration of chosen enzymes in the liver and muscles. The designated study groups differ in terms of diet used prior to and following surgery. It was assumed that following a bariatric operation the switch from a regular to an high fat (HF) diet and from an HF to a regular diet might be made. Then, we assessed the effect of the DJOS surgery together with control diet (CD), and an HF diet, in advance of and following surgery, on the glucose metabolism, measured using the enzymes mentioned above.

## Materials and Methods

### Animals and Diets

All experimental procedures were approved by the Ethical Committee for Animal Experimentation (58/2014). The methodology of DJOS was previously described by Stygar et al. (2018; Karcz et al., 2013). Briefly, 56 male Sprague-Dawley rats (Charles River Breeding Laboratories, Wilmington, MA, United States) aged 7 weeks, 200 ± 7 g, were housed at a 12-h light–dark cycle, 22°C and 40–60% humidity. All rats had free access to water and rat food. The control group was maintained on a professional, regular diet [Provimi Kliba AG, Kaiseraugst, Switzerland, 24% protein, 4.9% fat, 7% crude ashes, 4.7% crude fibre, lysine (13.6 g/kg), calcium (12 g/kg), methionine (4.5 g/kg), and phosphorus (8.3 g/kg)]. Obesity was induced by keeping the animals on a high-fat diet (HF; 23.0 kJ/g, 59% fat, 27% carbohydrate, and 14% protein [EF RAT (E15744) Ssniff Spezialdiäten GmbH] for the period of 2 months before surgery. Animals maintained on the HF diet were pair-fed (kcals) with the animals exposed to an *ad libitum* control diet. All rats fasted overnight before surgery.

### Experimental Design

**FIGURE 1:**
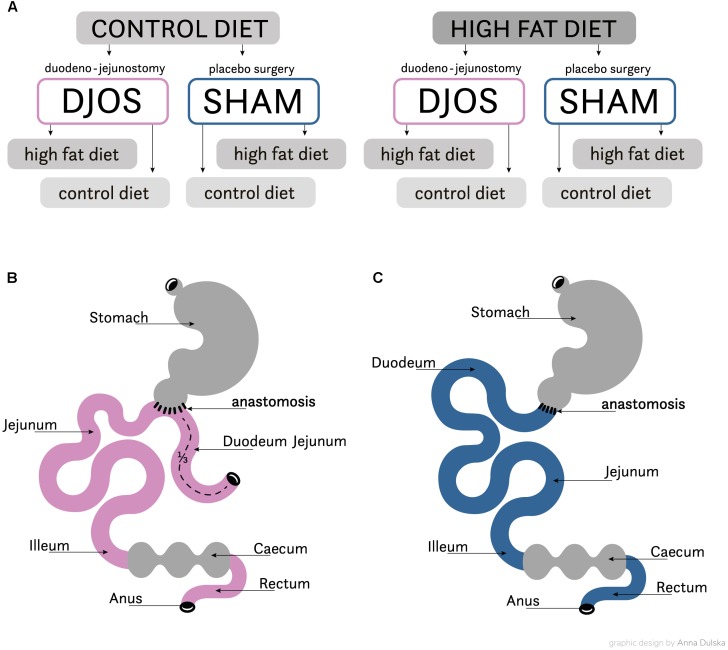
**(A)** Scheme of experimental groups. **(B)** Schematic illustration of DJOS and **(C)** SHAM surgery, respectively.

After 1 week of acclimatisation the rats were allocated to their experimental groups: high fat (HF, *n* = 28) and control (CD, *n* = 28). The total length of the experiment was 16 weeks. For 8 weeks before and after the DJOS and SHAM surgery the animals were maintained on their allocated diets. The initial part of the procedure, prior to surgery, included 8 weeks of maintenance on experimental diets. Following this, both groups were further split into subgroups, which then underwent two separate types of surgery: Sham (*n* = 14) and DJOS (*n* = 14) surgery was conducted in each group (Figure 1A). For the second stage of the experiment, through the 8 weeks following the surgery, seven animals from the DJOS and SHAM groups were maintained on the same diet as prior to surgery taking place (HF/DJOS/HF, HF/SHAM/HF, CD/DJOS/CD, CD/SHAM/CD), and a further seven from each group had an altered diet (HF/DJOS/CD, HF/SHAM/CD, CD/DJOS/HF, CD/SHAM/HF; Figure 1A). The number of rats included in the research was minimalised in consideration of the ‘3Rs’ for the humane treatment of animals (Russell and Burch, 1959). In the HF/SHAM/CD subgroup six out of seven rats were still alive at the end of the experiment and in the other subgroups the survival rate was 100%.

### Experimental Procedures

The DJOS was performed according to Karcz et al. (2013) methodology, described in the aforementioned study (Stygar et al., 2018). To perform DJOS, the animals were anaesthetised with 2% isoflurane (AbbVie Deutschland GmbH and Co. KG, Ludwigshafen, Germany) and oxygen flow at 2 l/min under spontaneous breathing. Analgesia with xylazine (5 mg/kg, i.p.; Xylapan, Vetoquinol Biovet, Poland) and antibiotic prophylaxis with gentamicin (4 mg/kg, KRKA, Poland) were applied. In order to gain abdominal access, a midline incision of 3–4 cm was performed, and the total length of the small intestine was determined (Figure 1B). The stomach was separated from the duodenum at the point just below the pylorus and the position of anastomosis was defined as being at 1/3 of the total small bowel length. The jejunum was anastomosed via end-to-side duodeno-enterostomy in order to restore the physiological conduit of the food passage, excluding the duodenum and parts of the small intestine. The remaining duodenal stump was closed using PDS 6/0 (Ethicon). Mesenteric openings were closed with PDS 6/0 (Ethicon). In the SHAM operated animals, reanastomosis of the gastrointestinal tract was performed at the corresponding sites where enterotomies were performed for the duodenojejunostomy, thereby maintaining continuity of the food passage through the bowel (Figure 1C). For DJOS and SHAM protocols, postoperative analgesia was performed using carprofen (4 mg/kg, sc; Rimadyl, Pfizer, Switzerland) for three consecutive days after the surgery.

### Tissue Collection

For tissue collection, anaesthesia was induced and maintained using isoflurane 2% and oxygen flow at 2 l/min breathing rate. Liver and muscle tissues were harvested and the animals were euthanised. Tissue samples were prepared by homogenisation and sonification (15 s) on ice in a tissue cell lysis buffer containing protease inhibitors (Gold Biotechnology, United States). After that, the homogenates were centrifuged at 15,000 × *g* for 15 min at 4°C. Homogenates were snap frozen in liquid nitrogen and stored at −80°C until further analysis.

### Concentration of Enzymes in Liver and Muscle Tissues

Liver and muscle tissue concentrations of glycogen 152 synthase kinase 3 alfa (GSK-3α, product no. SEA630Ra), glycogen phosphorylase (PYGM, product no. SEA848Ra; PYGL product no. SEA849Ra), and phosphofructokinase 1 (PFK-1 product no. SED406Ra) in liver and muscle were assessed in duplicate by the immunoenzymatic method with the commercially available ELISA kit (USCN Life Science Inc., United States) after an optimisation procedure. To assess the validity and predict tissue concentration of chosen parameters in 100 mg of tissue, the optimal sample dilutions for their particular experiments of ELISA bioassay were preliminarily determined.

### Statistical Analysis

Statistical analysis was performed using STATISTICA 12.5 PL (StatSoft, Cracow, Poland). Statistical significance was set at a *p*-value below 0.05. All tests were two-tailed. Interval data were expressed as mean value ± standard deviation in the case of normal distribution or as median/lower–upper quartile range in the case of data with skewed or non-normal distribution. Distribution of variables was evaluated by the Shapiro–Wilk test and the quantile-quantile plot, homogeneity of variances was assessed by the Levene test. For comparison of data the two-way parametric ANOVA with *post hoc* contrast analysis were used. In case of skewed data distribution logarithmic transformation was done before analysis.

## Results

The results of body weight change, OGTT, AUC_OGTT_ and insulin levels, after DJOS and SHAM surgery, in all experimental groups were previously presented by Stygar et al. (2018). Briefly, a change of diet from CD to HF and *vice versa* after DJOS and SHAM-type of surgery led to impaired insulin tolerance. This process was not observed in the study groups maintained on the same type of diet before and after surgery such as HF/HF and CD/CD. The analysis of OGTT, showed that the animals which underwent DJOS surgery expressed improved glucose tolerance in HF/HF and CD/CD study groups. For those groups, the 30-and 60-min plasma glucose levels were significantly decreased when compared to SHAM animals. DJOS animals exhibited significantly reduced AUC_OGTT_ and ameliorated glucose tolerance in the groups maintained on the same diet before and after the surgery HF/HF and CD/CD. DJOS surgery together with type of diet used in the experiment influenced the level of insulin in all subjects. A proper dietary pattern implemented pre and post DJOS surgery was pivotal for body weight, glucose tolerance, and insulin resistance.

Tissue concentrations of GSK-3α, PFK and PYGM, PYGL in DJOS and SHAM operated groups after long-term maintenance on HF and CD and mixed HF/CD and CD/HF eating patterns are shown in Table 1.

**Table 1:** Tissue concentrations of **GSK-3α, PYGM, PYGL, PFK** in liver and/or soleus muscle tissue of DJOS and SHAM operated groups after long-term maintenance on HF and CD and mixed HF/CD and CD/HF eating patterns.

	DJOS				SHAM				p ANOVA		
	HF/HF	HF/CD	CD/HF	CD/CD	HF/HF	HF/CD	CD/HF	CD/CD	Group	Op.	Int.
**Muscle**											
GSK-3α (ng/ml/mg w.t.)	12.9 ± 6.9	30.7 ± 9.4	5.0 ± 2.8	7.5 ± 2.9	18.5 ± 7.8	19.8 ± 9.7	6.5 ± 4.5	15.5 ± 5.9	<0.001	0.602	<**0.01**
PYGM (ng/ml/mg w.t.)	222.0 ± 35.5	194.4 ± 20.9	209.5 ± 24.1	211.3 ± 42,9	216.7 ± 21.2	208.0 ± 27.4	191.4 ± 18.8	210.7 ± 45.1	0.415	0.775	0.682
PFK (ng/ml/mg w.t.)	137.1 ± 36.2	27.6 ± 16.1	71.7 ± 18.8	63.1 ± 31.6	34.6 ± 11.4	30.8 ± 17.5	118.2 ± 48.7	35.7 ± 22.1	<**0.001**	<**0.05**	<**0.001**
**Liver**											
GSK-3α (ng/ml/mg w.t.)	144.1 ± 46.7	155.7 ± 36.4	233.0 ± 34.5	107.2 ± 37.4	56.7 ± 24.7	72.0 ± 27.6	147.5 ± 31.5	109.3 ± 24.1	<**0.001**	<**0.001**	<**0.01**
PYGL (ng/ml/mg w.t.)	200.5 ± 113.6	210.8 ± 107.6	301.7 ± 73.2	134.0 ± 92.6	204.4 ± 52.5	225.3 ± 39.5	348.7 ± 85.1	319.8 ± 82.2	<**0.01**	<**0.05**	<**0.05**
PFK (ng/ml/mg w.t.)	203.7 ± 56.4	347.0 ± 43.0	240.8 ± 51.0	212.2 ± 96.9	278.4 ± 133.4	248.6 ± 49.1	241.0 ± 41.6	188.7 ± 48.9	<**0.05**	0.582	0.054

*Descriptive statistics and results of two-way analysis of variance. Op., operation type; Int., interaction between group and operation type. Mean ± standard deviation or median (lower–upper quartile). Statistical significance for all obtained values was set at a p < 0.05. All values below 0.05 are shown in bold.*

Table 2 presents results of multiple comparisons in contrast analysis of DJOS and SHAM operated groups in relation to diet used before and after surgery. Column one presents a comparison between DJOS and SHAM surgery associated with different diets, column two shows comparisons between dietary groups of DJOS operated animals, and column three presents comparisons between dietary groups of SHAM operated animals.

**Table 2:** Results of multiple comparisons in contrast analysis of DJOS and SHAM operated groups in relation to diet used before and after surgery.

*Post hoc*	DJOS vs. SHAM				DJOS						SHAM					
	1: HF/HF	2: HF/CD	3: CD/HF	4: CD/CD	1 vs. 2	1 vs. 3	1 vs. 4	2 vs. 3	2 vs. 4	3 vs. 4	1 vs. 2	1 vs. 3	1 vs. 4	2 vs. 3	2 vs. 4	3 vs. 4
**Muscle**																
GSK-3α (ng/ml/mg w.t.)	0.154	<0.01	0.692	<**0.05**	<**0.001**	<**0.05**	0.171	<**0.001**	<**0.001**	0.505	0.746	<**0.01**	0.437	<**0.01**	0.273	<**0.05**
PYGM (ng/ml/mg w.t.)	–	–	–	–	–	–	–	–	–	–	–	–	–	–	–	–
PFK (ng/ml/mg w.t.)	<**0.001**	0.855	<**0.01**	0.102	<**0.001**	<**0.001**	<**0.001**	**0.018**	<**0.05**	0.615	0.818	<**0.001**	**0.945**	<**0.001**	0.765	<**0.001**
**Liver**																
GSK-3α (ng/ml/mg w.t.)	<**0.001**	<**0.001**	<**0.001**	0.913	0.570	<**0.001**	0.064	<**0.001**	<**0.05**	<**0.001**	0.435	<**0.001**	<**0.01**	<**0.001**	0.061	0.055
PYGM (ng/ml/mg w.t.)	0.936	0.776	0.335	<**0.001**	0.839	<**0.05**	0.176	0.080	0.137	<**0.05**	0.667	<**0.01**	<**0.05**	<**0.05**	0.057	0.553
PFK (ng/ml/mg w.t.)	0.082	<**0.05**	0.998	0.578	<**0.01**	0.380	0.840	<**0.05**	<**0.01**	0.497	0.481	0.377	<**0.05**	0.856	0.160	0.219

*Column one presents a comparison between DJOS and SHAM surgery associated with different dietary patterns, column two shows comparisons between dietary groups of DJOS operated animals, and column three presents comparisons between dietary groups of SHAM operated animals. Statistical significance for all obtained values was set at a p < 0.05. All values below 0.05 are shown in bold.*

### GSK-3α Muscle

**FIGURE 2:**
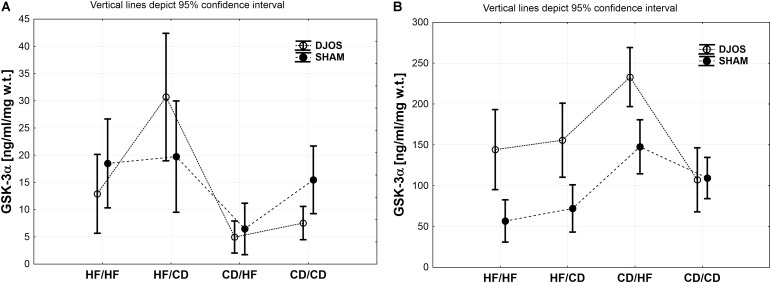
**(A)** GSK-3α (ng/ml/mg w.t.) muscle concentration in groups subjected to different dietary patterns, according to the DJOS and SHAM operation type. **(B)** GSK-3α (ng/ml/mg w.t.) liver concentration in groups subjected to different dietary patterns, according to the DJOS and SHAM operation type.

Comparing DJOS and SHAM-type of surgery, the same pattern in the GSK-3α concentration in the muscle tissue was observed. Nevertheless, GSK-3α muscle concentration was significantly higher for the DJOS group when compared to SHAM in HF/CD and CD/CD groups (Figure 2A and Tables 1, 2).

Taking into consideration DJOS procedure, the level of GSK-3α concentration after DJOS surgery was significantly lower in the group of animals kept on HF/HF diets before and after surgery in relation to HF/CD and higher in comparison to the CD/HF group (Figure 2A and Table 2). A significant increase in the concentration of GSK-3α was observed in the group kept on HF/CD when compared to CD/HF and CD/CD (Figure 2A and Table 2).

For the SHAM surgery group, statistically significant differences between CD/HF and the other following experimental groups were observed (Figure 2A and Table 2).

### GSK-3α Liver

Studied DJOS and SHAM surgeries and diets showed an impact on the GSK-3α concentration in the liver. For DJOS and SHAM surgery the same pattern of GSK-3α liver concentration was observed. A significantly higher concentration of GSK-3α in the liver tissue of DJOS operated rats in comparison to SHAM surgery was observed for animals handled on an HF diet before and/or after surgery in relation to the CD/CD group (Figure 2B and Tables 1, 2).

Taking into consideration DJOS procedure, the higher GSK-3α liver concentration was observed in the groups kept on an HF diet before and/or after surgery in comparison to CD/CD study groups. The DJOS operated animals kept on a CD/HF diet showed a higher GSK-3α liver concentration in comparison with other DJOS operated study groups (Figure 2B and Table 2).

After SHAM-type of surgery, the GSK-3α liver level was statistically higher in HF/HF vs. CD/HF; HF/HF vs. CD/CD; HF/CD vs. CD/HF groups (Figure 2B and Table 2). GSK-3α liver concentration of the HF/HF group was significantly lower in comparison to the CD/HF and CD/CD, and HF/CD was significantly lower in comparison to the CD/HF group (Figure 2B and Table 2).

### PFK Muscle

**FIGURE 3:**
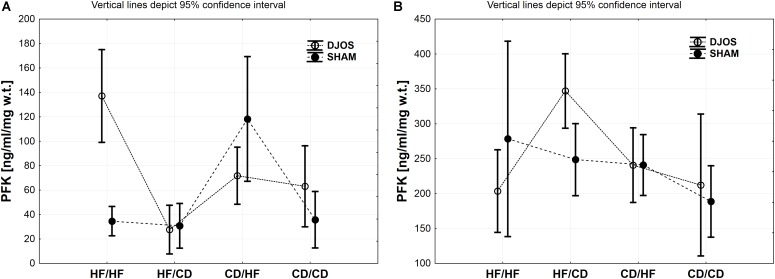
**(A)** PFK (ng/ml/mg w.t.) muscle concentration in groups subjected to different dietary patterns, according to the DJOS and SHAM operation type. **(B)** PFK (ng/ml/mg w.t.) liver concentration in groups subjected to different dietary patterns, according to the DJOS and SHAM operation type. Statistical significance was set at a *p* < 0.05. Vertical lines depict 95% confidence interval. DJOS, duodenal-jejunal omega switch surgery; HF, high fat diet; CD, control diet; HF/HF, CD/HF, HF/CD, CD/CD, type of diet 8 weeks before/8 weeks after surgery.

Diet, more than the DJOS and SHAM surgery, influenced PFK concentration in the soleus muscle. In the DJOS operated group, HF diet used before and after surgery significantly increased PFK liver levels in comparison to SHAM surgery and the CD/HF group (Figure 3A and Tables 1, 2).

After DJOS surgery, the HF/HF group showed significantly higher concentration of PFK in muscle tissue in comparison with other studied groups (Figure 3A and Table 2). The lowest concentrations of PFK were observed in the HF/CD group when compared to CD/HF and CD/CD groups of DJOS operated animals (Figure 3A and Table 2).

The highest impact on PFK muscle concentration in SHAM operated rats had a change of the diet after surgery from CD to HF diet in relation to other SHAM studied groups (Figure 3A and Table 2).

### PFK Liver

Significantly higher PFK liver concentration was observed in the HF/CD DJOS operated group when compared to SHAM-type of surgery (Figure 3B and Tables 1, 2).

In the DJOS operated rats, PFK liver concentration in the HF/CD group was significantly higher when compared to other DJOS studied groups (Figure 3B and Table 2).

In the SHAM operated rats, different types of diet had the highest impact on PFK concentration in the HF/HF vs. CD/CD groups (Figure 3B and Table 2).

### PYGM Muscle

**FIGURE 4:**
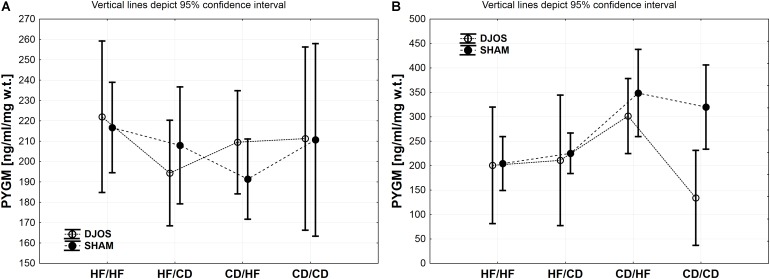
**(A)** PYGM (ng/ml/mg w.t.) muscle concentration in groups subjected to different dietary patterns, according to the DJOS and SHAM operation type. **(B)** PYGL (ng/ml/mg w.t.) liver concentration in groups subjected to different dietary patterns, according to the DJOS and SHAM operation type. Statistical significance was set at a *p* < 0.05. Vertical lines depict 95% confidence interval. DJOS, duodenal-jejunal omega switch surgery; HF, high fat diet; CD, control diet; HF/HF, CD/HF, HF/CD, CD/CD, type of diet 8 weeks before/8 weeks after surgery.

The descriptive statistics and two-way analysis of variance showed no significant differences between the analysed groups in terms of PYGM concentration in muscles after DJOS and SHAM surgery. Thus, the multiple comparisons in contrast analysis for this enzyme were not performed (Figure 4A and Table 2).

### PYGL Liver

A similar trend in the PYGL concentration in liver tissue of DJOS and SHAM operated rats was observed. The type of surgery applied had an impact on the PYGL levels in the liver of rats from CD/CD group of DJOS vs. SHAM operated animals. Nevertheless, this relation was not observed in other studied groups, where the impact of diet was stronger (Figure 4B and Tables 1, 2).

Change of the diet from CD before to HF after DJOS surgery increased PYGL levels in comparison to HF/HF and CD/CD diet conditions (Figure 4B and Table 2).

The CD/HF group of SHAM operated rats showed significantly higher levels of PYGL in comparison to HF/HF and HF/CD (Figure 4B and Table 2). A high fat obesogenic diet applied before and after SHAM surgery resulted in a significantly lower level of PYGL in comparison to the regular diet CD/CD group (Figure 4B and Table 2).

## Discussion

In the presented work, we studied the effect of manipulation of different dietary patterns, applied 8 weeks before and 8 weeks after DJOS or SHAM surgery, and conducted on Sprague-Dawley rats. In the described study design, the levels of selected glycolytic enzymes in the soleus muscle and liver tissue were analysed. The main findings of this work are: (i) The type of diet applied before and after surgery had a stronger impact on the levels of selected glucose metabolic enzymes than DJOS or SHAM surgery; (ii) The impact of DJOS and SHAM surgery was visible for GSK-3α and PYGL concentration in liver tissue but not in soleus muscle tissue; (iii) The type of surgery had an impact on liver GSK-3α concentration in all studied groups except the CD/CD group, where the impact of diet was stronger; (iv) DJOS surgery influenced the level of PYGL in the livers of rats maintained on the CD/CD diet but not from other groups.

Under conditions of insulin resistance, the activity of the GSK-3α in skeletal muscle, and liver is enhanced (Henriksen, 2010). However, its inhibition leads to an increase in glycogen synthesis and insulin sensitivity (Henriksen and Dokken, 2006; Lee and Kim, 2007; Rayasam et al., 2009). In our experiment, the type of diet showed a stronger impact on the concentration of GSK-3α than surgery in soleus muscle tissue of DJOS and SHAM operated animals. GSK-3α concentration was higher than in the control, in the groups maintained on the HF diet before/after surgery. GSK-3 has been shown to be elevated in the skeletal muscle of type 2 diabetic subjects (Nikoulina et al., 2001), and in the animal models of insulin resistance (Eldar-Finkelman et al., 1999; Dokken et al., 2005). In this work, the animals maintained on CD before and after the surgery, showed a lower concentration of GSK-3α in the soleus muscle tissue in comparison with the other experimental groups. The same trend was observed in the group of animals maintained on the CD diet before and HF after the DJOS and SHAM surgery. Diet and type of surgery influenced the liver concentration of GSK-3α, which was higher after SHAM surgery in comparison to DJOS. In our previous study, we have reported increased insulin resistance in the groups of animals maintained on the different diets, HF/CD and CD/HF, before and after DJOS surgery. A change of dietary pattern after surgery impaired insulin tolerance and GIP effect more than maintaining the animals on the same type of diet pre and post surgery.

The DJOS operated animals from the HF/HF and CD/CD, but not from CD/HF and HF/CD groups, exhibited significantly reduced AUC_OGTT_, and therefore ameliorated glucose tolerance (Stygar et al., 2018). Despite the type of analysed surgeries, a change of the diet, from CD to HF and HF to CD, increased liver concentration of GSK-3α. Insulin resistance is associated with hepatic glycogen synthesis. The conversion of glucose into hepatic glycogen is a primary mechanism of reducing plasma glucose concentrations following a nutrients intake (Wang et al., 2017). We propose that, a change of diet after 8 weeks, and then the intake of different types of nutrients for the following 8 weeks, had an effect on the biochemical pathways, which was expressed in the GSK-3α levels and therefore, decreased hepatic glycogenesis by suppressing GS activity. We can conclude that the selection and change of dietary pattern has a strong impact on GSK-3α muscle and liver levels. HF increased GSK-3α levels despite the DJOS or SHAM surgery, nevertheless the DJOS shows a positive impact on the normalisation of GSK-3α liver tissue concentration in comparison to SHAM surgery.

Numerous studies implicate that diabetes reduces PFK-1 activity in different cell tissues (Sochor et al., 1987; Rossi et al., 1990; Khoja and Salem, 1991; Morimoto et al., 1995). PFK-1 can be considered a potential target for the treatment of obesity. It was found that mice deficient in the M isozyme of PFK-1 had significantly decreased fat stores (Getty-Kaushik et al., 2010). This leads to the conclusion, that inhibition of muscle PFK-1 could improve physiological processes and support the treatment of obesity, diabetes and metabolic syndrome (Malina et al., 2014). In this study, we observed that type of diet used before and after surgery had a stronger impact on the PFK-1 soleus muscle and liver concentration than the surgery. The combination of DJOS surgery and HF/HF diet significantly increased PFK concentration when compared to SHAM operated animals. It was also the highest concentration when compared to other DJOS operated study groups. The change from highly obesogenic HF diet into a less proinflammatory and lighter in terms of energy intake CD decreased the levels of PFK-1 when compared to HF/HF. Moreover, a change of the diet from CD to HF in SHAM operated animals significantly increased PFK-1 levels in the muscle tissue. We can conclude that an HF diet and the change of the dietary patterns to an HF diet significantly increased PFK-1 muscle levels in comparison to the control group, both in DJOS and SHAM operated animals. The similar trend after DJOS and SHAM procedures was observed in the liver tissue, where change of diet or the presence of HF influenced and/or increased the profile of PFK-1 concentration in comparison to control groups. These results implicate that a combination of dietary patterns and a change of the diet *per se* but not DJOS surgery has a crucial, beneficial impact on PFK-1 levels in the soleus muscle and liver tissue. The liver produces glucose mainly by two biochemical pathways: gluconeogenesis, which is *de novo* synthesis of glucose, and by glycogenolysis, the breakdown of glycogen by liver phosphorylase. It has also been demonstrated that the inhibition of GP is involved in promoting glycogen synthesis in the liver. Thus GP, and especially its liver isoform, is a potential therapeutic target in maintaining control of blood glucose levels and minimising the debilitating effects of diabetes (Rath et al., 2000; Donnier-Marechal and Vidal, 2016). In this study, the diet had stronger impact on PYGL liver concentration than the type of surgery applied. A change in the type of diet from CD to HF increased the level of PYGL, both in DJOS and SHAM operated groups. The additive effect of DJOS surgery and CD were observed in the group of animals kept on CD before and after the surgery. The lower level of this enzyme may be understood as a means of controlling/reducing glucose levels in circulating blood.

## Conclusion

We conclude that DJOS surgery has a weaker impact on the tissue concentrations of GSK-3α, PYGL, and PFK-1 than dietary patterns used in the experiment. The dietary patterns applied before and after surgery had a significant impact on enzyme concentrations than DJOS surgery, where the strong, deleterious effect of an HF diet was observed. Moreover, a change of the diet *per se* also showed a negative impact on the enzymes tissue concentration.

## Ethics Statement

This study was carried out in accordance with Directive 2010/63/EU of the European Parliament and of the Council of 22 September 2010 on the protection of animals used for scientific purposes. The protocol was approved by the Local Ethical Commission for Experiments on Animals in Katowice Medical University of Silesia in Katowice.

## Author Contributions

DS and KK conceived the idea of this experiment. DS, BS-P, TS, and EO maintained animals. DS conducted surgery. DS, DA, and JJ worked on the manuscript. EC and DS analysed data and performed statistical analysis. DS and BB carried out analysis. All authors gave their final approval of the submitted and published version.

## Conflict of Interest Statement

The authors declare that the research was conducted in the absence of any commercial or financial relationships that could be construed as a potential conflict of interest.
